# Regional, racial, gender, and tumor biology disparities in breast cancer survival rates in Africa: A systematic review and meta-analysis

**DOI:** 10.1371/journal.pone.0225039

**Published:** 2019-11-21

**Authors:** Paddy Ssentongo, Joseph A. Lewcun, Xavier Candela, Anna E. Ssentongo, Eustina G. Kwon, Djibril M. Ba, John S. Oh, Forster Amponsah-Manu, Alicia C. McDonald, Vernon M. Chinchilli, David I. Soybel, Daleela G. Dodge

**Affiliations:** 1 Center for Neural Engineering, Department of Engineering, Science and Mechanics, The Pennsylvania State University, Pennsylvania, United States of America; 2 Department of Surgery, Penn State Hershey College of Medicine and Milton S. Hershey Medical Center, Hershey, Pennsylvania, United States of America; 3 Department of Public Health Sciences, Penn State Hershey College of Medicine and Milton S. Hershey Medical Center, Hershey, Pennsylvania, United States of America; 4 Department of Surgery, Eastern Regional Hospital, Koforidua, Ghana; Duke University, UNITED STATES

## Abstract

**Background:**

The survival rates from breast cancer in Africa are poor and yet the incidence rates are on the rise. In this study, we hypothesized that, in Africa, a continent with great disparities in socio-economic status, race, tumor biology, and cultural characteristics, the survival rates from breast cancer vary greatly based on region, tumor biology (hormone receptor), gender, and race. We aimed to conduct the first comprehensive systematic review and meta-analysis on region, gender, tumor-biology and race-specific 5-year breast cancer survival rates in Africa and compared them to 20-year survival trends in the United States.

**Methods:**

We searched MEDLINE, EMBASE, and Cochrane Library to identify studies on breast cancer survival in African published before October 17, 2018. Pooled 5-year survival rates of breast cancer were estimated by random-effects models. We explored sources of heterogeneity through subgroup meta-analyses and meta-regression. Results were reported as absolute difference (AD) in percentages. We compared the survival rates of breast cancer in Africa and the United States.

**Findings:**

There were 54 studies included, consisting of 18,970 breast cancer cases. There was substantial heterogeneity in the survival rates (mean 52.9%, range 7–91%, *I*^**2**^ = 99.1%; p for heterogeneity <0.0001). Meta-regression analyses suggested that age and gender-adjusted 5-year survival rates were lower in sub-Saharan Africa compared to north Africa (AD: –25.4%; 95% CI: –34.9 - –15.82%), and in predominantly black populations compared to predominantly non-black populations (AD: –25.9%; 95% CI: 35.40 - –16.43%). Survival rates were 10 percentage points higher in the female population compared to male, but the difference was not significant. Progesterone and estrogen receptor-positive breast cancer subtypes were positively associated with survival (*r* = 0.39, p = 0.08 and *r* = 0.24, p = 0.29 respectively), but triple-negative breast cancer was negatively associated with survival. Survival rates are increasing over time more in non-black Africans (55% in 2000 versus 65% in 2018) compared to black Africans (33% in 2000 versus 40% in 2018); but, the survival rates for Africans are still significantly lower when compared to black (76% in 2015) and white (90% in 2015) populations in the United States.

**Conclusion:**

Regional, sub-regional, gender, and racial disparities exist, influencing the survival rates of breast cancer in Africa. Therefore, region and race-specific public health interventions coupled with prospective genetic studies are urgently needed to improve breast cancer survival in this region.

## Background

Across the globe, breast cancer is the most common cancer affecting women and the leading cause of cancer–related death.[1] Of the 17.5 million new cancer cases diagnosed globally in 2015, 2.4 million were breast cancer, accounting for 53,000 deaths. Of these, 44,000 (~2%) breast cancers occurred in men.[2] While the incidence of breast cancer is highest in high-income countries (HICs), the incidence in low and middle-income countries (LMICs) has been rising.[3, 4] In addition to the significant mortality, in 2015, breast cancer was responsible for 15 million disability-adjusted life-years (DALYs) worldwide. Breast cancer mortality rates are substantially higher in LMICs than in HICs. For example, in 2018, age-standardized mortality rates due to breast cancer in LMICs was 14.9 per 100,0000 females compared to 11.6 per 100,000 females in HICs.[5] While breast cancer outcomes in HICs have improved significantly over the last several decades in LMICs, breast cancer mortality still mirrors that of the pre-screening era (prior to 1976) in the United States. This difference has in significant part been attributed to late-stage at diagnosis and limited access to treatment.[1]

Of the 2.4 million new cases of breast cancer worldwide diagnosed in 2015, approximately 300,000 (13%) occurred on the African continent. Incidence and mortality rates due to breast cancer in Africa are increasing.[3, 6] For example, between 1993–2007, the annual percentage change in the incidence rate of breast cancer in Uganda was 5.2% and the annual percentage change in the mortality rate in Mauritius between 1993–2012 was 3.5%.[7] Survival rates vary depending on the geographical location, stage of the disease, tumor biology, and access to care.[8–10] Understanding the causes of these variations in survival rates of breast cancer and delineating the factors that are associated with poor outcomes are key to improving survival. To our knowledge, survival rates of breast cancer for the entire continent of Africa have not been examined systematically. In this study, we review the published literature on survival rates of breast cancer in African countries. The main objective of this review is to ascertain the current and historical survival rates of breast cancer patients on the African continent and delineate any possible sources of heterogeneity between studies. These findings may help define future strategies that will be key to improving survival in populations with breast cancer on the African continent.

## Methods

### Search strategy and selection criteria

We used a study protocol (**S1 Text**) based on the PRISMA guidelines. We searched three databases (MEDLINE, EMBASE, and Cochrane Library) to identify all studies published before October 17, 2018, which reported on the survival rate of primary invasive breast cancer in women and men in the whole of Africa. The United Nations (UN) classification was used to define Africa into sub-Saharan and north Africa. African countries were grouped according to sub-regions (i.e., north, south, east, west, and central Africa). We search words using keyword-based on Medical Subject Headings (MeSH) with the following search terms: “survival rate”, AND “Africa” AND “breast cancer”, “breast neoplasm”, “breast carcinoma”, “breast sarcoma”, “breast tumor”, “breast tumour”, or “breast malignancy*”, (S1 Table). No restrictions were imposed on the gender, age at diagnosis, ethnicity, the clinical setting of diagnosis (public or private) or language of the publication.

We selected articles according to the following steps. First, title and abstract were reviewed to identify records that were deemed eligible for inclusion. We excluded articles that restricted inclusion to survival less than five years, paper reviews, and studies that were case reports or case series. Next, full-text articles were retrieved and reviewed to confirm eligibility.

### Quality assessment and data extraction

Four authors (PS, JL, XC, and AS) independently extracted and reviewed data from eligible full-text articles, using an adapted version of a standard data entry electronic form. Disagreements between extractors were discussed until a consensus was reached by the majority rule decision. The 5-year overall survival rate of the study population was extracted from each eligible paper using the survival rate values reported. If not reported, we extracted the survival rates from the published Kaplan Meier curves using the method described Tierney and colleagues (2007).[11] Data on the country of the study, date of publication, study design, study population, year of diagnosis, and the mean or median age at the time of diagnosis were also extracted. When a study did not report on racial composition, their population was assumed to have the racial composition of their country of origin’s population. Studies done in north Africa countries were presumed to be a predominantly non-black population. When available, data were extracted on survival rate by biologic prognostic markers including hormone receptor status (estrogen and progesterone), human epidermal growth factor receptor 2 (HER2) status and survival rates by early-stage (I/II) and late-stage (III/IV). Next, three authors (JL, PS and EK) independently assess the quality of the articles using an adapted version of a standardized form, which was developed using an approach similar to that of the Cochrane collaboration (see details in the S2 Text). Data from three domains was considered: detailed demographic information, stratified reporting of survival, and overall sample size. Based on the sum of those domains, a quality score ranging from 0–24 (low to high quality) was given to each paper.

### Data analysis

The primary outcome was the estimation of the overall 5-year survival probability of breast cancer in Africa. The *metaprop* function from R package *meta* was used to calculate the pooled effect estimates of 5-year survival rates using random effect models.[12] We used the DerSimonian-Laird (DL) estimator method to estimate the pooled between-study variance.[13] Results were graphically displayed in form of forest plots.

We assessed between-study heterogeneity using *I*^2^ statistic, expressed as % (low (25%), moderate (50%), and high (75%) and Cochrane’s *Q* statistic (significance level < 0.05).[14, 15] We conducted meta-regression analyses to investigate sources of variations. Regressors were: sub-Saharan versus north Africa, black versus non-black African, region (east, west, north and south, and central Africa) and year of publication. The estimates were gender and age-adjusted. Study-level determinants of survival are expressed as absolute differences (AD) in the 5-year survival and reported in percentage. We assessed small study bias using funnel plots and the Egger test.[16]

We compared the 5-year overall survival in Africa to that of the USA. The analysis was stratified by race (white and black population in the USA and non-black and black population in Africa). For the USA survival rates, we used data from the Surveillance, Epidemiology, and End Results (SEER) database, which include information on all cases of invasive primary breast cancer in women from nine US population-based cancer registries.[17]

## Results

**Fig 1:**
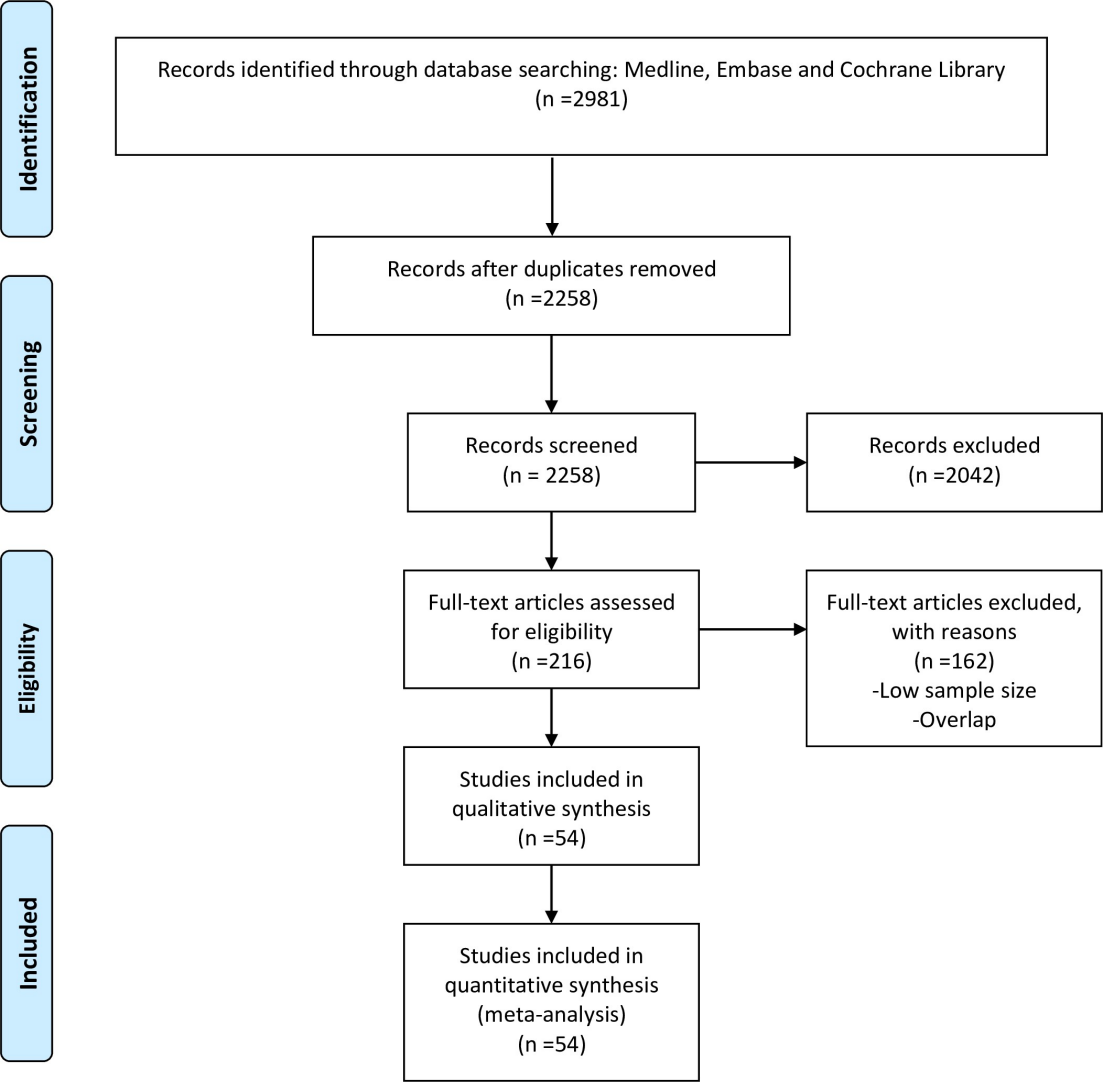
PRISMA flowchart of a systematic review of the breast cancer survival rate in Africa.

**Fig 2:**
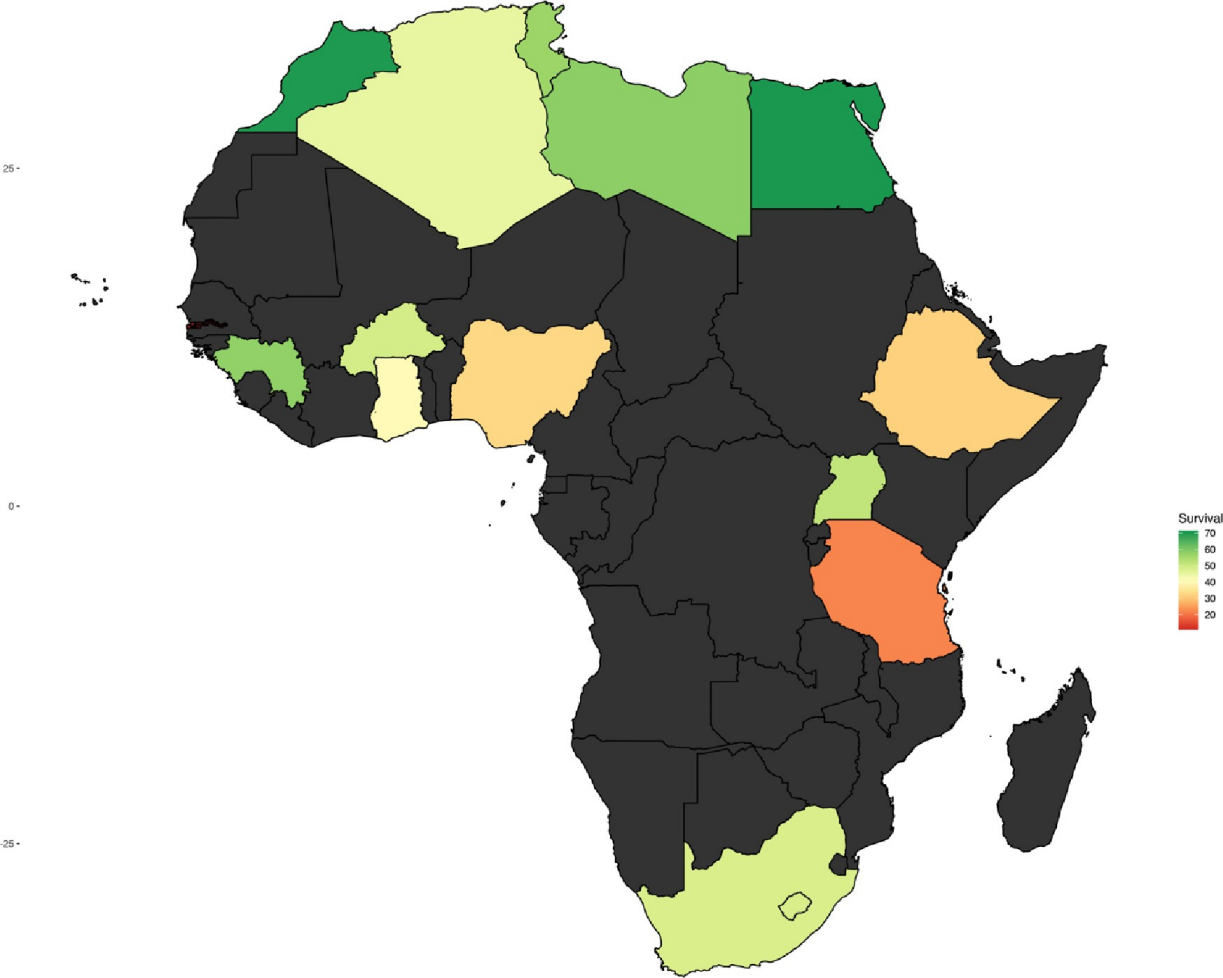
Survival rate (%) of breast cancer shaded by the country from which the papers used in the meta-analysis were published. No data in black shade.

There were 2,258 articles identified from our literature search after duplicate studies were excluded. Of these, 216 full-text articles were eligible based on the inclusion criteria (Fig 1). After full review of these papers, 54 were included in the final analysis representing 13 Africa countries, 8 of which were from sub-Saharan Africa (Fig 2). All references used in the meta-analysis and their details are given in S3 Table.

**Fig 3:**
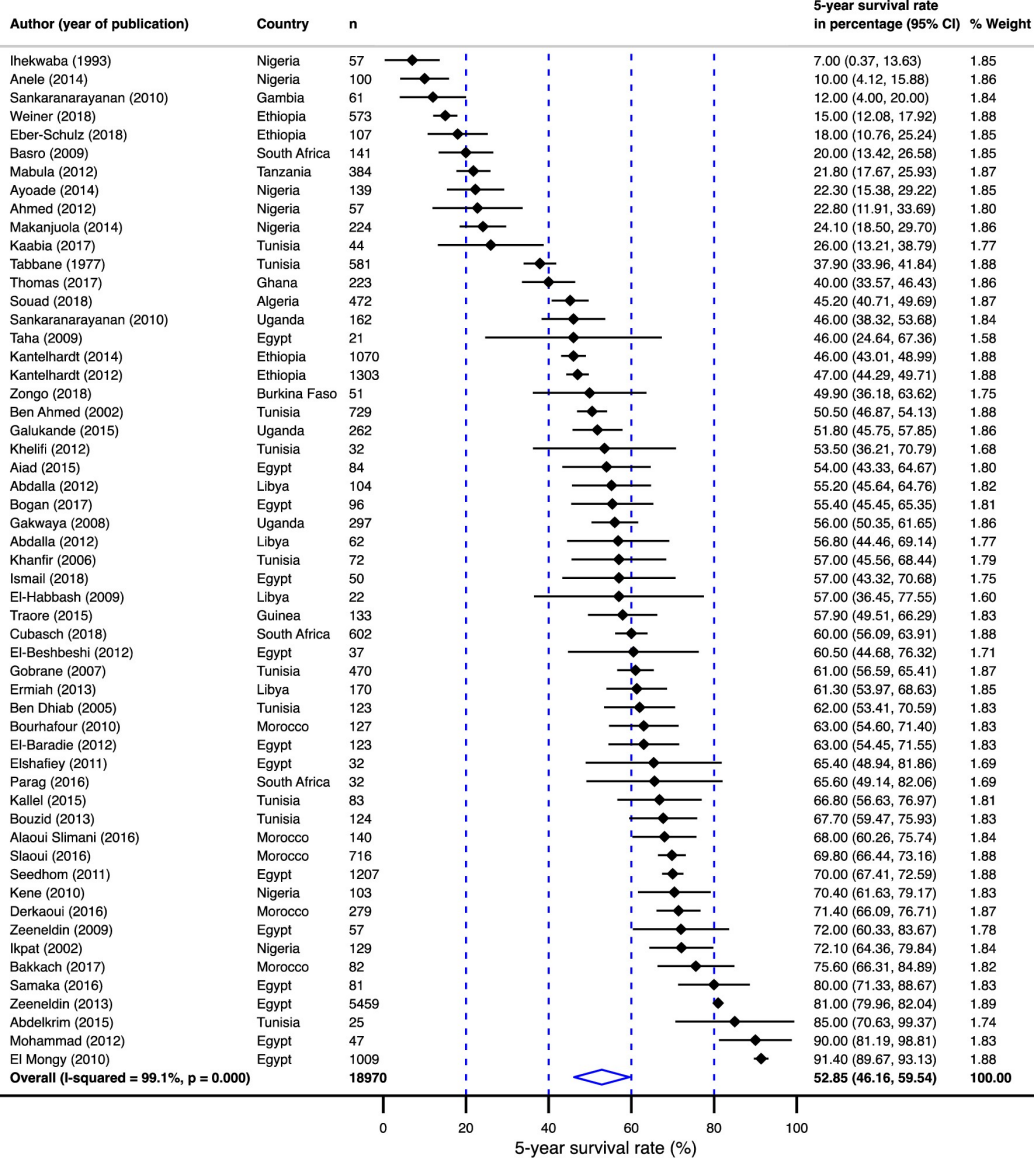
Pooled 5-year survival rate in breast cancer population in Africa.

Of the 54 papers included, there were a total of 18,970 patients with breast cancer, with a study sample size ranging from 21 to 5,459 (median = 433; Fig 3). Thirty-four studies (60%) were from north Africa (13,489 breast cancer patients). Of the 21 studies from sub-Saharan Africa, 7 (33%) were from Nigeria (806 patients with survival probability information), 4 studies (19%) were from Ethiopia (3,053 patients), 3 studies (14%) were from South Africa (775 patients), 3 studies (14%) were from Uganda (721 patients), and 1 (5%) study each from Algeria (472 patients), Burkina Faso (51 patients), Gambia (61 patients), Ghana (133 patients), and Tanzania (384 patients). The median age of breast cancer diagnosis was 48 years (range 14 to 96 years), and 25.5%, 29.1%, and 40% of the studies had a median age at breast cancer diagnosis of <45 years, 45–50 years, and 50 years or older, respectively. Three studies did not report a mean age at diagnosis or age range.

**Fig 4:**
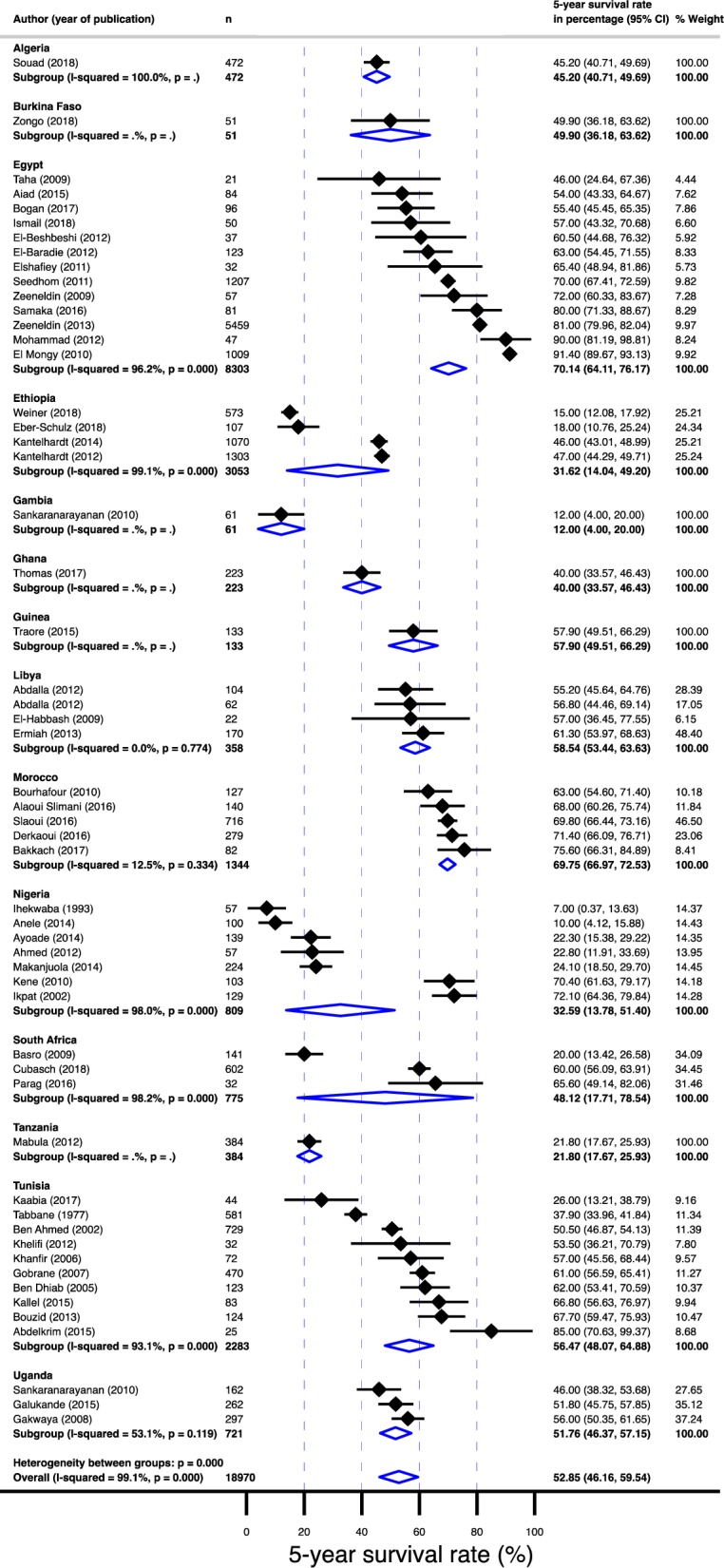
Pooled 5-year survival rate in breast cancer population in Africa stratified by country from which the papers used in the meta-analysis were published.

**Fig 5:**
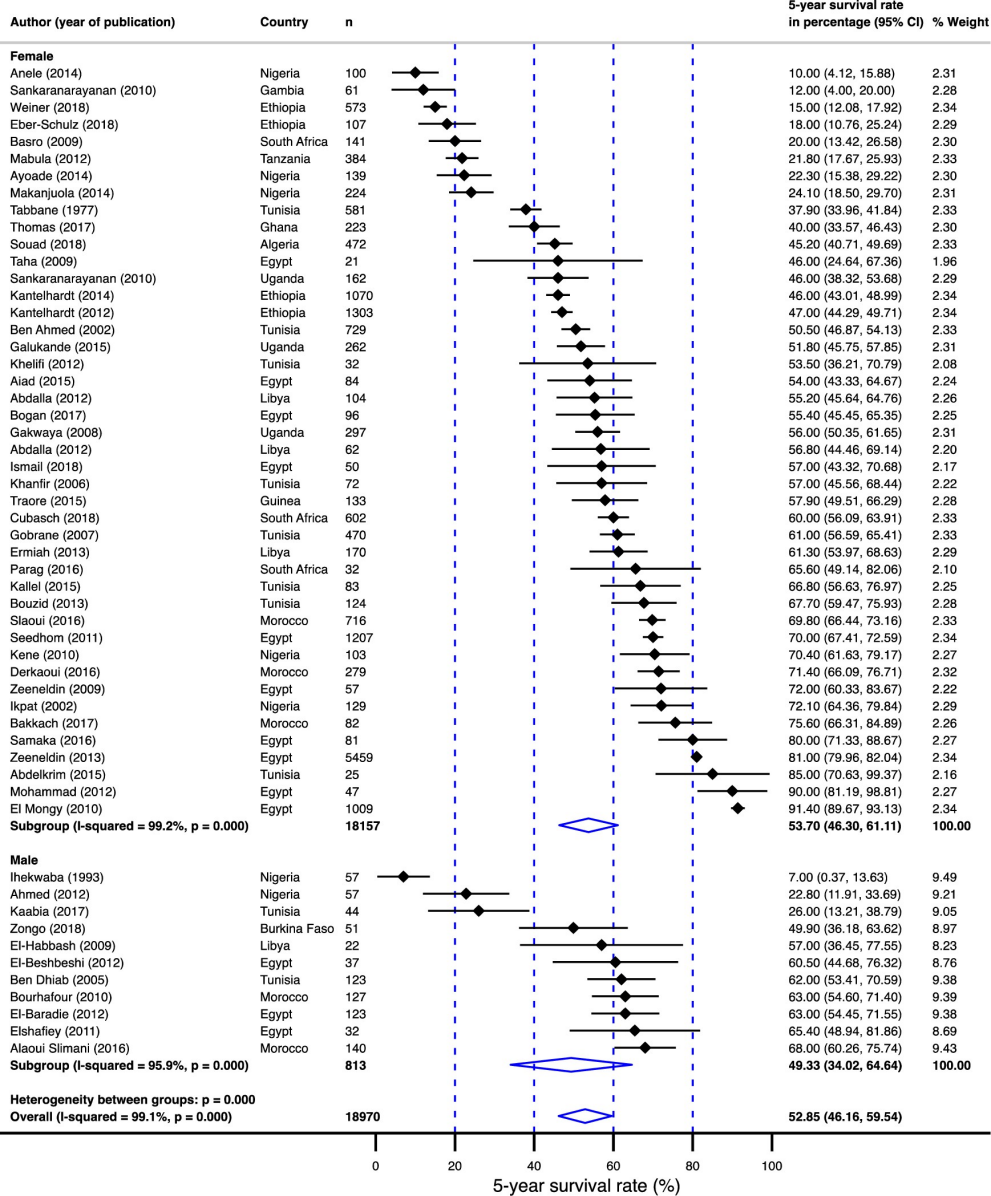
Pooled 5-year survival rate in breast cancer population in Africa stratified by gender.

Displayed in Fig 3 are 5-year survival rates by country. The overall 5-year survival rate reported was 52.9% (95% confidence interval [CI]: 46.2–59.5%). The survival rate ranged from 7% (95% CI: 2–17%) in Nigeria[18] to as high as 91% (95% CI: 89–93%) in Cairo, Egypt[19] (Fig 4). Between-study variation in the 5-year survival rates was high (*I*^2^ = 99.1%; p for heterogeneity <0.0001). Of the 54 studies evaluated, 19 (35%) (from western and eastern Africa) were done exclusively in the black population. When a study did not report on racial composition, their population was assumed to have the racial composition of their country of origin’s population. Eleven studies (20%) reported on breast cancers in exclusively male populations. The survival rates in males with breast cancer were 4% lower than the survival rates in female populations (Fig 5).

**Fig 6:**
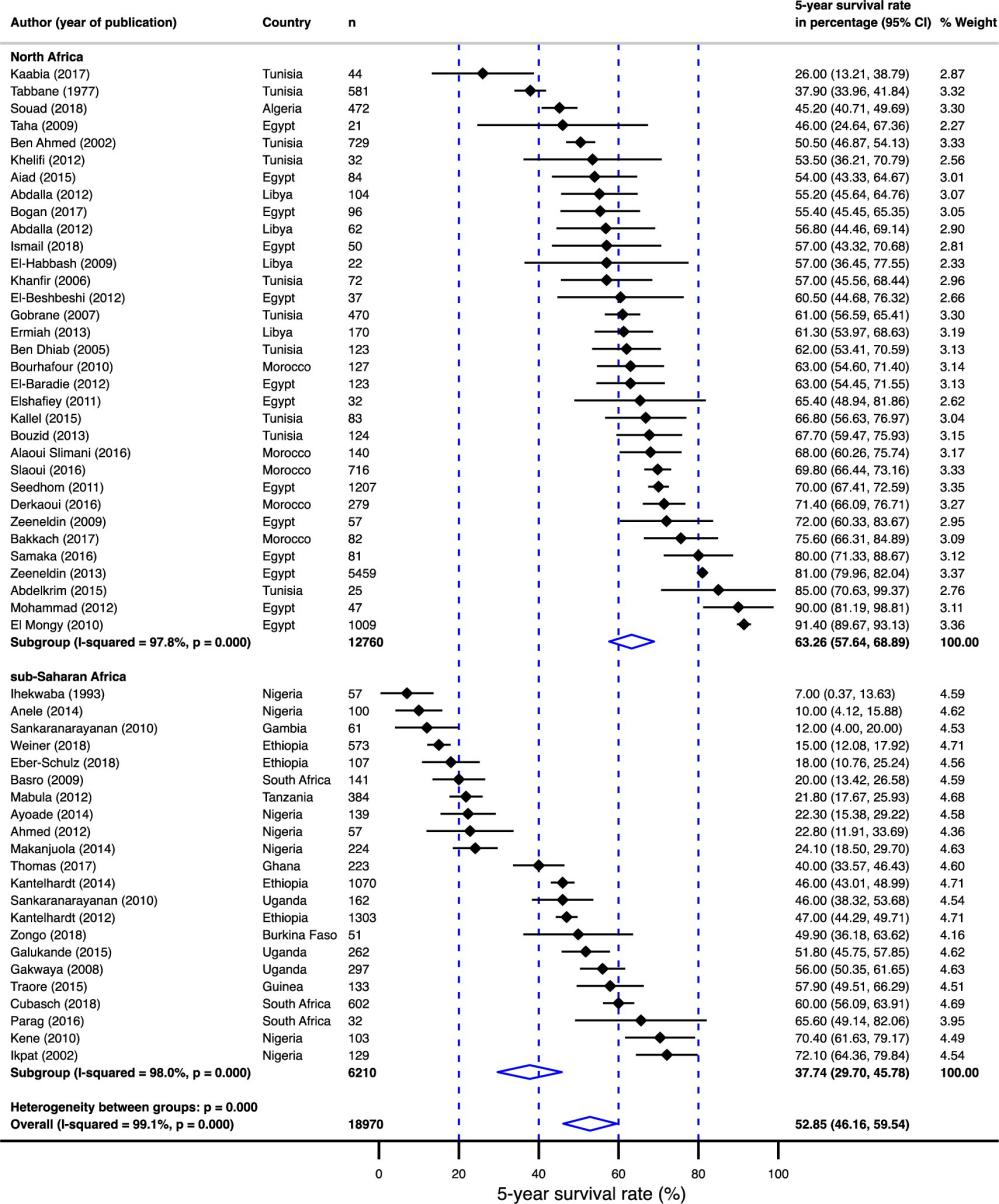
Pooled 5-year survival rate in breast cancer population in Africa stratified by region (sub-Saharan Africa vs. non-sub-Saharan Africa) from which the papers used in the meta-analysis were published.

**Fig 7:**
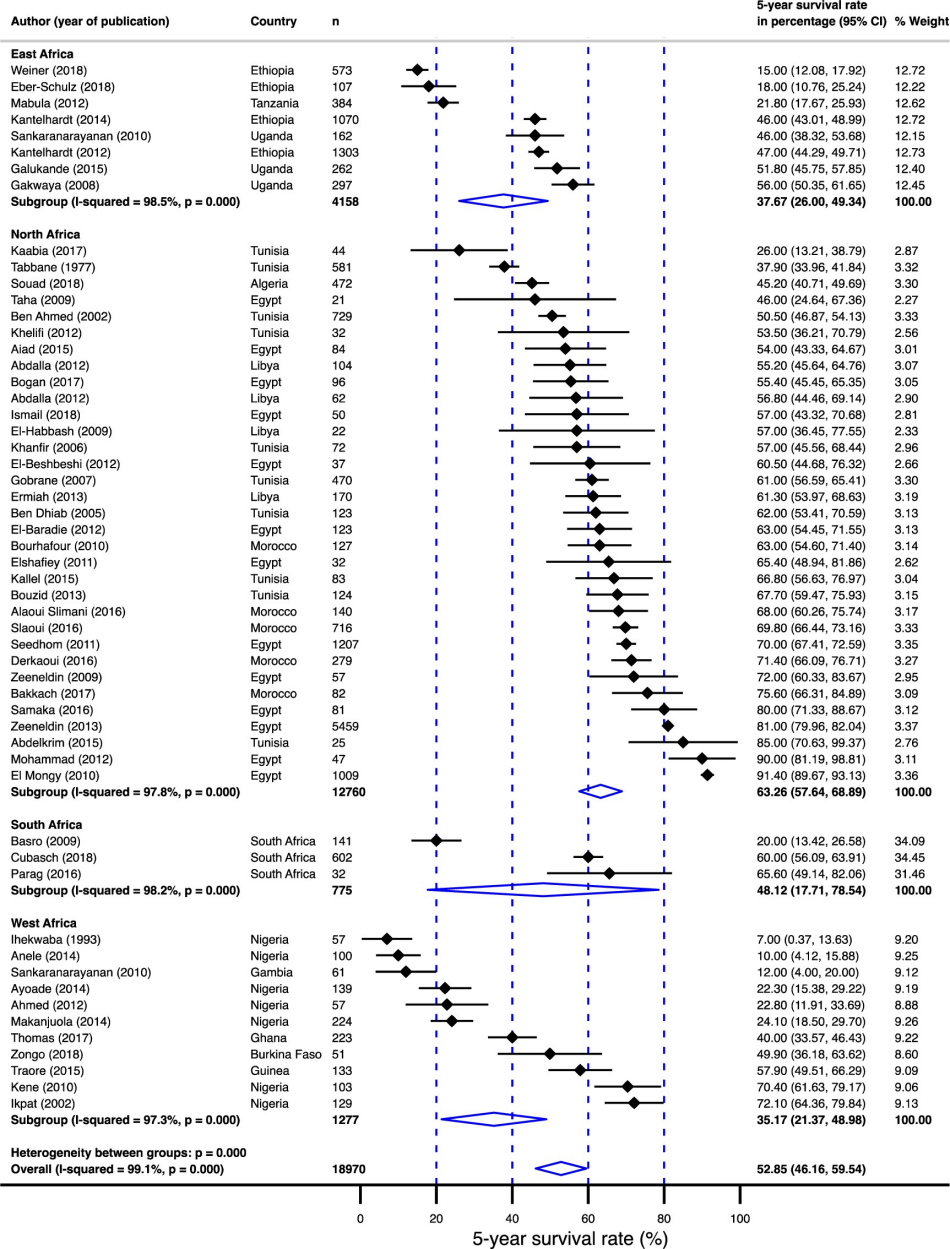
Pooled 5-year survival rate in breast cancer population in Africa stratified by sub-regions (East Africa, West Africa, South Africa and North Africa) from which the papers used in the meta-analysis were published.

**Fig 8:**
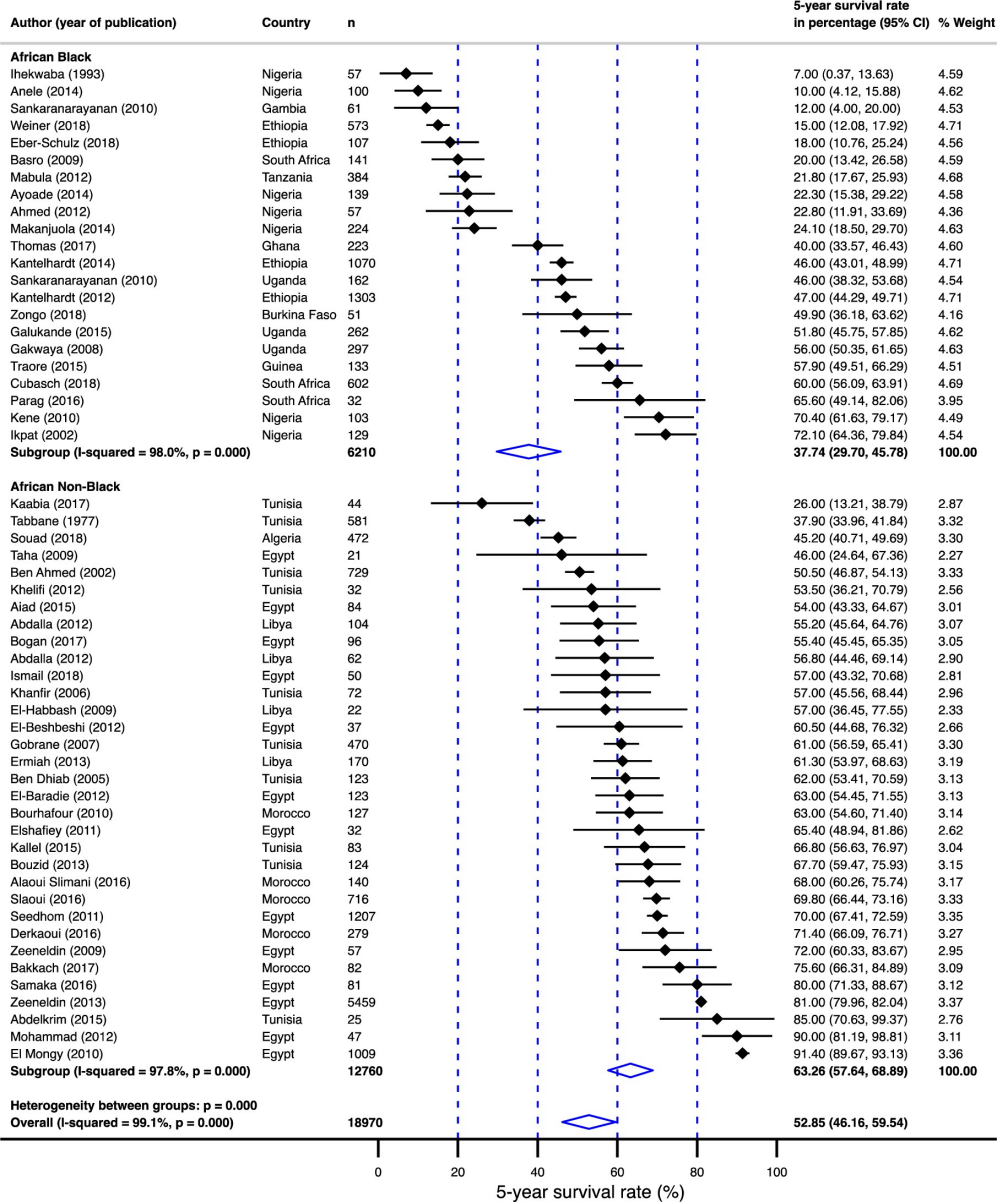
Pooled 5-year survival rate in breast cancer population in Africa stratified by black versus non-black race.

Summarized in Table 1 is the age and gender-adjusted meta-regression analysis. Survival rates were 25% lower (95% CI: –34.97 - –15.82%) for sub-Saharan Africa compared to north Africa (Fig 6). In addition, the survival rates were statistically significantly different between sub-regions; the survival rate was 28% lower (95% CI: –39.48 - –15.98%) for west Africa, 24% lower (95% CI: –38.24 - –9.65%) for east Africa and 22% lower (95%: CI: –46.74–2.35%) for south Africa compared to north Africa (Fig 7). Furthermore, compared to non-black populations, survival in the black population was 26% lower (95% CI: –35.40 - –16.43%; Fig 8). Although survival rates were 10 percentage points higher in female population compared to males, the difference was not significant. Finally, age did not contribute to the heterogeneity in the survival rates.

**Table 1:** Meta-regression analysis showing age and gender-adjusted absolute difference (%) in 5-year survival rate in breast cancer population in Africa.

Predictors of survival rates	Sample Size	Adjusted absolute difference in survival rate (%) (95% CI)	*p*-value
**Age (y)**	18,970	0.16 (-0.53, 0.84)	0.66
**Gender**			
Female	18,157	10.20 (-4.79, 25.18)	0.182
Male	813	Reference	
**Sub-Region**			
West Africa	1,277	-27.73 (-39.48, -15.98)	<0.001
East Africa	4,158	-23.94 (-38.24, -9.65)	0.001
South Africa	775	-22.20 (-46.74, 2.35)	0.076
North Africa	12,760	Reference	
**Region**			
Sub-Saharan	6,210	-25.40 (-34.97, -15.82)	<0.001
North Africa	12,760	Reference	
**Race**			
Black	5,608	-25.92 (-35.40, -16.43)	<0.001
Non-black	13,362	Reference	
**Publication Year**			
Above 2010	14,230	0.95 (-11.65, 13.54)	0.883
2010 and lower	4,890	Reference	

**Fig 9:**
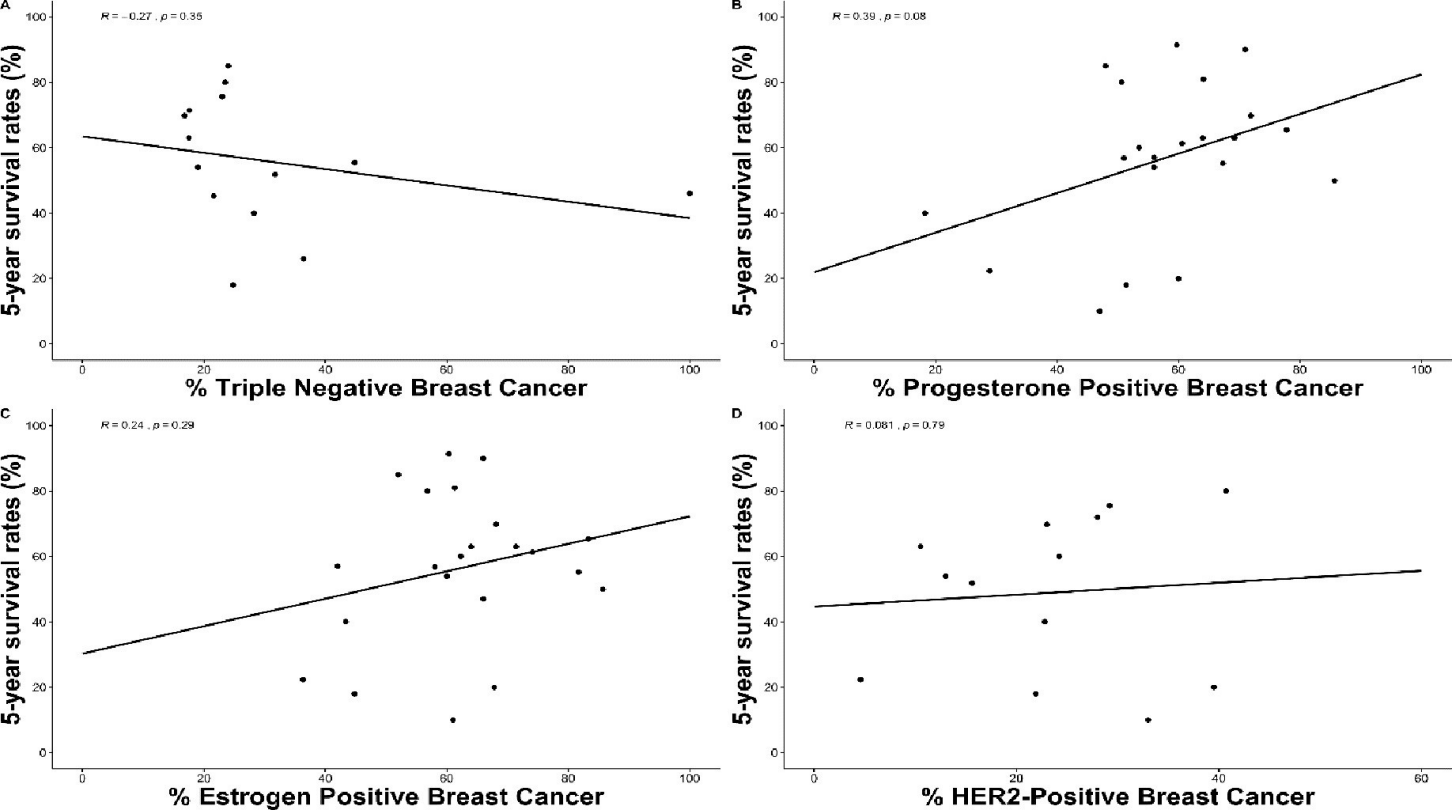
Correlation of survival rates in breast cancer patients and hormone receptor status.

Of the 22 studies that reported on tumor biology, triple-negative breast cancer (TNBC) was negatively correlated with 5-year survival rate (Pearson correlation coefficient *r* = –0.27, p = 0.35, n = 14 studies); and, positive progesterone receptor status was positively correlated with 5- year survival rate (*r* = 0.39, p = 0.08; n = 21 studies). However, these associations were not statistically significant. Similarly, positive estrogen receptor status (*r* = 0.24, p = 0.29; n = 22 studies) and HER-2 positive receptor status (*r* = 0.081, p = 0.79; n = 13 studies) had a non-significant positive correlation with survival rates, (Fig 9).

**Fig 10:**
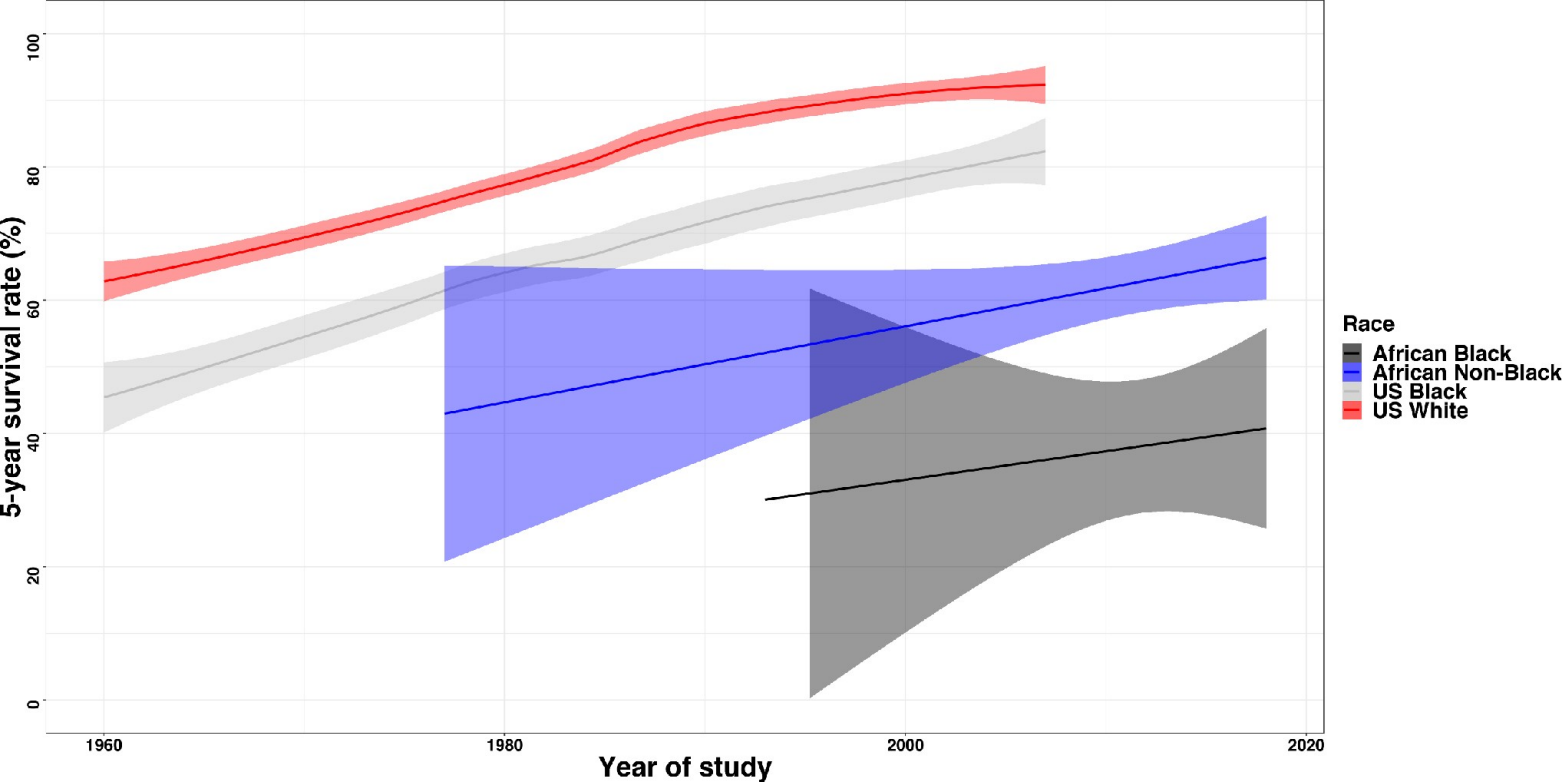
Trends in 5-year survival rate in the United States compared to Africa stratified by race (white vs. black in the US, and black and non-black in Africa). Bands are 95% confidence intervals.

Compared to the survival rates in the United States, the overall 5-year survival was lower on the African continent (Fig 10). The current survival rate of the black population on the African continent was 30% lower when compared to the black population in the United States.

## Discussion

We estimated the 5-year survival rates of breast cancer throughout the entire African continent. We compiled data from 54 studies consisting of 18,970 patients with breast cancer who had follow-up data available to determine survival probability. Our results suggest that exploring survival rates on a global or regional level could mask the unique differences of the disease burden within a sub-region level. The major finding is that, in African countries, 5-year survival rate following diagnosis of breast cancer is only 53%. In the present study, 5-year survival rates in sub-Saharan Africa were 26% lower than those of north Africa. It is worth noting that the majority of the population in sub-Saharan Africa is predominantly black, as opposed to majority non-black populations found in north Africa.[20] The western region of sub-Saharan Africa had the worst 5- year survival of 35%, followed by the eastern region of 38%, and finally the southern region of 48%. In part, this could be explained by the socio-economic and government health spending variation among these regions. The southern region has a stronger economy and higher government health spending than either eastern or western Africa as evidenced by a substantially higher government health spending per capita in 2017 of $594.0 in South Africa compared to $93 in Ghana, $17 in Ethiopia, $ 59 in Tanzania and $22 in Uganda.[21] The status of a national economy correlates directly with other factors that are known to influence survival such as stage at diagnosis, access to care, psychosocial well-being and nutrition.[22, 23] Although breast cancer mortality across the African continent is much higher than has been observed in Europe and North America,[5] our findings identified a slow but steady increase in survival rates in both black and non-black populations in Africa. This observed trend is encouraging and could be explained by a trend toward earlier stage at diagnosis. For example, a case series of 1,200 patients with breast cancer showed a decrease in the frequency of late-stage III/IV cancers in South Africa, from 66% in 2006–07 to 46% in 2010–12.[24] This decrease could be caused, in part, by increased awareness and acceptance of the treatment for breast cancer. It also highlights the success of campaigns targeted at raising awareness and reducing associated stigma–both that breast cancer is a curse and the stigma associated with mastectomy.[25] In middle-income countries, improved diagnostics such as the use of ultrasound or mammography may also have contributed to this improvement.[26] Most importantly improved access to care and treatment have impacted breast cancer survival.

In sub-Saharan Africa, lower levels of breast cancer awareness and other barriers to access to health care such as longer distances to health-care facilities are key drivers of late-stage presentation and subsequent poor survival rates observed in this region.[27–30] However, the reasons for the marked heterogeneity of survival between populations in sub-Saharan Africa versus north Africa, in blacks versus non-blacks and in males versus females, are not entirely clear. Although most patients were aged 35–49 years at diagnosis (approximately 10–15 years younger than patients in developed countries), [31] we did not find a strong association between age at diagnosis and overall survival. The young age at presentation calls attention to two major issues. First, it reflects the younger age structure of the African population, stemming from the higher fertility rates of the population and yet a shorter life expectancy. Second, there are inherent biological differences in the breast cancers seen in young black populations, as indicated by the higher prevalence of TNBC reported in this population.[10, 27, 32]. It should be noted however that the high TNBC breast tumor biology seen in Africa could be an artifact. As discussed in a review by Eng and colleagues,[33] tumor biology assessment is affected by factors including inadequate or prolonged fixation, non-standardized histological preparation and assessment, delay from tissue biopsy to histological preparation and evaluation. These delays may increase the risk of protein degradation, and therefore, these specimens tend to yield lower ER+ and PR+ frequency estimates due to antigen degradation as a function of time.

The 5-year survival rate has been rising steadily in the USA between 1973 and 2015, from 50% to 90% in white populations and 40% to 76% in black populations (Fig 10). By contrast, most study-specific estimates of survival rates in black sub-Saharan African population have remained well below 50% between1970s and 2015, significantly lower than that of non-black population in north Africa. In other words, the current survival rates of the black sub-Saharan African population are still lower than that of the USA five decades ago despite improved access to care and breast cancer treatment.

Major strengths of this review is the large sample size of approximately 20,000 men and women with breast cancer in Africa. However, there were limitations. First, due to scarcity of published data, we could only include studies from 20 of the 54 Africa countries. Second, published literature only included patients who presented at health-care facilities, predominantly tertiary centers, and as such, this review may not be a truly representative sample of all patients with breast cancer in Africa. Distance to a health care facility remains a key driver for late-stage presentation and poor treatment adherence across the African continent.

This review demonstrates that the 5-year overall survival rates of breast cancer in the black populations of Africa around 2015 were lower than those of both black and white populations in the USA 50 years ago. Therefore, early diagnosis of breast cancer through breast cancer awareness efforts coupled with improved treatment should be one of the breast cancer control strategies deployed in this region. A recent Breast Health Global Initiative consensus statement provides a template for improving breast cancer outcomes in LMICs.[34] Studies from Uganda and Ethiopia—both sub-Saharan African countries—demonstrated that there are improved survival rates in populations that are diagnosed at earlier stages.[9, 35] Dispelling the stigma associated with a diagnosis of breast cancer and measures leading to earlier diagnosis, when coupled with timely and appropriate treatment, can improve breast cancer survival in Africa in the future.

## Conclusion

In this first-of-the-kind systematic review and meta-analysis of breast cancer survival rates in the entire continent of Africa, we showed that there are regional, sub-regional, gender, and racial disparities in survival rates. Therefore, region and race-specific public health interventions coupled with prospective genetic studies urgently are needed to improve breast cancer survival.

## Supporting information

S1 Text Systematic review protocol. (DOCX)

S2 Text Grading system for the quality of the papers included in the analysis. (DOCX)

S1 Fig Funnel plot assessing small study bias. (DOCX)

S1 Table Literature search strategy. (DOCX)

S2 Table PRISMA 2009 checklist. (DOCX)

S3 Table Details of each study. (DOCX)
