# Supplementary material for: Regional, racial, gender, and tumor biology disparities in breast cancer survival rates in Africa: A systematic review and meta-analysis
Source: PLoS One. 2019 Nov 21;14(11):e0225039. doi: 10.1371/journal.pone.0225039 (PMC6872165; doi:10.1371/journal.pone.0225039)
Supplement: S1 Text — (DOCX) [file pone.0225039.s001.docx]

**Regional, racial, gender, and tumor biology disparities in breast cancer survival rates in Africa: a systematic review and meta-analysis**

**S1 Text. Systematic Review Protocol**

**Regional, racial, gender, and tumor biology disparities in breast cancer survival rates in Africa: a systematic review and meta-analysis**

Paddy Ssentongo^1,2,3^, Joseph Lewcun^2^, Xavier Candela^2^, Anna E. Ssentongo^2,3^, Eustina G. Kwon^2^, Djibril M. Ba^3^, John S. Oh^2^, Forster Amponsah-Manu^2,4^, Alicia C. McDonald^3^, Vernon M. Chinchilli^3^, David I. Soybel^2^, Daleela G. Dodge^2^

**Background**

In 2015, 2.4 million new cases of breast cancer were diagnosed worldwide^1^, and approximately 53,000 persons died of this disease. It was responsible for 15 million disability-adjusted life-years (DALYs). Breast cancer is the most common cancer affecting women and the leading cause of cancer–related deaths in this group globally.^2^ Of the 2.4 million new cases, approximately 300,000 (13%) occurred in Africa. Breast cancer mortality rates are higher in low-income countries (LMICs) than in high-income countries (HICs), such as those in sub-Saharan Africa, despite their lower incidence because of late-stage at diagnosis and limited access to treatment.^2^ Breast incidence and mortality rates in Africa are increasing, and the survival rates depend on the geographical location, stage of the disease, tumor characteristics and access to care. In order to optimize survival in Africa, it is critical to understand variations in the survival rates of breast cancer across Africa. Variations in survival rates of breast cancer across Africa have been previously reported in individual settings^3-7^ but have not been examined systematically across Africa. In this study, we seek to review the published literature on survival rates of breast cancer in African countries, examine trends over time, and investigate sources of variations. The findings may help to identify groups at increased risk of poor survival and guide the prioritizing of resource allocation for early detection and treatment of this disease.

**Objectives**

The objective of this review is to ascertain the 5- year survival rates of breast cancer patients in Africa. Specific aims are:

1. To provide an overview of 5-year survival rates of breast cancer across Africa;
2. To delineate sources of heterogeneity of in survival rates of breast cancer across Africa
3. To compare the trend in breast cancer survival rates of black and non-black populations of Africa to that of the US population.

**Search Strategy**

*Inclusion criteria*

• Reported on the survival rates of primary invasive breast cancer in women in any African country

• Conducted and published before October 17, 2018;

• Published in any language

*Exclusion criteria*

• Not conducted in humans;

• Meeting abstracts, review papers, and commentaries;

**Database searches**

The databases to be searched will include:

• EMBASE

• Medline

• Cochrane Library

**Search Terms**

We will use Keywords and Medical Subject Headings (MeSH) to search the databases. We will used the following search terms: “breast cancer” and “Africa”. We will develop a full list of search terms.

**Title and abstract screening**

We will search the databases listed above. The citations will be downloaded into the Endnote software. We will exclude duplicate articles. Three reviewers will independently screen titles and abstracts and will document, with reasons, studies that are excluded from the review.

**Full-text screening and data extraction**

We will extract data from eligible the papers identified during the abstract screening step. We will extract the following information: country of study, region of study, race (non-black versus black), study design, study population and, mean/median age at the time of diagnosis, tumor’s receptor status.

**Assessment of Methodological Quality of the Papers**

Three reviewers independently will assess the quality of the papers included in the review using a standardized form. The quality assessment form capture three broad categories of items. These categories will examine information and selection bias. We will allocate a score to each item within the three domains. (0 to 4). Higher scores represent better quality of the reviewed paper.

**Data Analysis**

We will use R Statistical Software for analysis. The primary outcome will be the 5- year survival rates, defined as the 5-year probability of survival. We will extract survival information from the manuscript, either stated or using extracting it from Kaplan Meir curves. The R package will be used to graphically display population-specific survival probability and estimated pooled probabilities using random effect models. We will employ I^2^-statistics and the P-value for heterogeneity (Cochrane’s Q statistic) test to examine between-study heterogeneity. Meta-regression analysis will be performed to identify independent sources of heterogeneity such as country/region, gender, age and year of publication.

We will assess small study bias using funnel plots and the Egger test. We will use the US Surveillance Epidemiology and End Results (SEER) database to analyse the 5- year survival rates in the US, comparing it with that of Africa.

**References**

1. Fitzmaurice C, Allen C, Barber RM, et al. Global, regional, and national cancer incidence, mortality, years of life lost, years lived with disability, and disability-adjusted life-years for 32 cancer groups, 1990 to 2015: a systematic analysis for the global burden of disease study. *JAMA oncology* 2017; **3**(4): 524-48.

2. Torre LA, Islami F, Siegel RL, Ward EM, Jemal A. Global cancer in women: burden and trends. AACR; 2017.

3. Galukande M, Wabinga H, Mirembe F. Breast cancer survival experiences at a tertiary hospital in sub-Saharan Africa: a cohort study. *World journal of surgical oncology* 2015; **13**(1): 220.

4. Gakwaya A, Kigula-Mugambe J, Kavuma A, et al. Cancer of the breast: 5-year survival in a tertiary hospital in Uganda. *British journal of cancer* 2008; **99**(1): 63.

5. Cubasch H, Dickens C, Joffe M, et al. Breast cancer survival in Soweto, Johannesburg, South Africa: A receptor-defined cohort of women diagnosed from 2009 to 11. *Cancer epidemiology* 2018; **52**: 120-7.

6. Gado N, Kamal M, Mousa S, et al. Survival outcomes in Egyptian elderly patients with breast cancer: single institute experience. *European Journal of Cancer* 2017; **72**: S10.

7. Slaoui M, Mouh FZ, Ghanname I, Razine R, El Mzibri M, Amrani M. Outcome of breast cancer in moroccan young women correlated to clinic-pathological features, risk factors and treatment: a comparative study of 716 cases in a single institution. *PloS one* 2016; **11**(10): e0164841.
