# Supplementary material for: Regional, racial, gender, and tumor biology disparities in breast cancer survival rates in Africa: A systematic review and meta-analysis
Source: PLoS One. 2019 Nov 21;14(11):e0225039. doi: 10.1371/journal.pone.0225039 (PMC6872165; doi:10.1371/journal.pone.0225039)
Supplement: S2 Text — (DOCX) [file pone.0225039.s002.docx]

**S2 Text. Grading system for the quality of the papers included in the analysis**

**Detailed demographic information: Max points: 11**

- Average age stated: 0, 1
- Gender mentioned: 0, 1
- Study design mentioned: 1, 2
  - Retrospective cohort: 1
  - Prospective cohort: 2
- Race mentioned: 0,1
- Stage or grade percentages reported: 0,1
- Treatment modalities available at facility mentioned: 0,1
- Receptor status percentages reported: max points 4
  - ER: 0, 1
  - PR: 0, 1
  - Her2/Neu: 0, 1
  - TNBC: 0, 1

**Stratified reporting of survival: Max Points: 7**

- 5-year survival reported: max points 2
  - Kaplan-Meier estimation: 1
  - Through direct 5-year patient follow-up: 2
- Survival by stage or grade reported: 0, 1
- Survival by receptor status reported: max points 4
  - ER: 0, 1
  - PR: 0, 1
  - Her2/Neu: 0, 1
  - TNBC: 0, 1

**Sample size: Max points: 6**

- Overall sample size: max points 3
  - less than 50: 1
  - 50-150: 2
  - greater than 150: 3
- Percentage of patients with successful follow-up: max points 3
  - Less than 50%: 1
  - 50-75%: 2
  - Greater than 75%: 3

**Total max score from three domains: 24**
