# Supplementary figures and images for: Regional, racial, gender, and tumor biology disparities in breast cancer survival rates in Africa: A systematic review and meta-analysis
Source: PLoS One. 2019 Nov 21;14(11):e0225039. doi: 10.1371/journal.pone.0225039 (PMC6872165; doi:10.1371/journal.pone.0225039)

**S1 Figure. Funnel plot assessing small study bias**


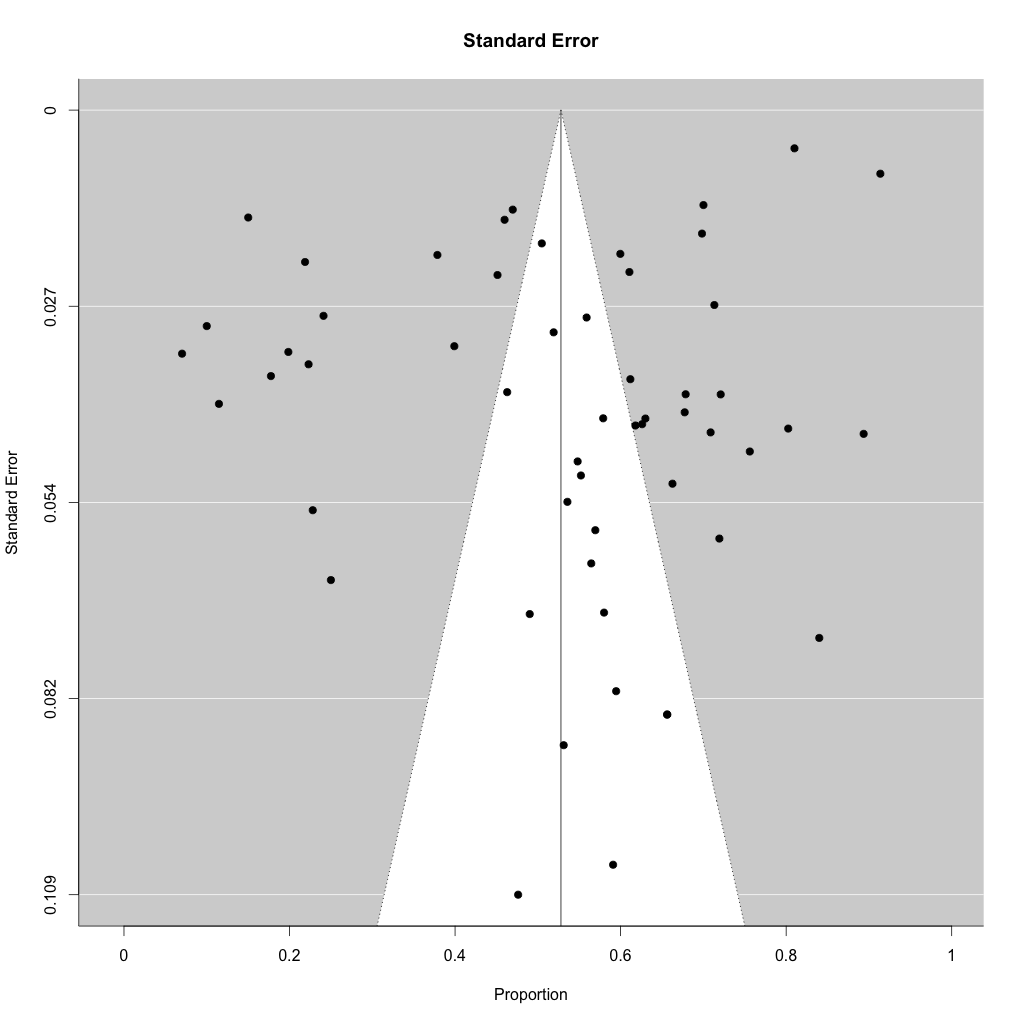

Supplement: S1 Fig — (DOCX) [file pone.0225039.s003.docx]
