# Supplementary material for: Regional, racial, gender, and tumor biology disparities in breast cancer survival rates in Africa: A systematic review and meta-analysis
Source: PLoS One. 2019 Nov 21;14(11):e0225039. doi: 10.1371/journal.pone.0225039 (PMC6872165; doi:10.1371/journal.pone.0225039)
Supplement: S1 Table — (DOCX) [file pone.0225039.s004.docx]

| **Database** | **Search Terms** |
| --- | --- |
| **Medline** | 1. (breast cancer* or breast neoplasm* or breast carcinoma* or breast sarcoma* or breast  tumor* or breast tumour* or breast malignanc*).mp. [mp=title, abstract, original  title, name of substance word, subject heading word, keyword heading word, protocol supplementary  concept, rare disease supplementary concept, unique identifier]  2. (survival rate or mortality rate).mp. [mp=title,  abstract, original title, name of substance word, subject heading word, keyword heading word,  protocol supplementary concept, rare disease supplementary concept, unique identifier]  3. (Africa or sub Saharan Africa or Angola or Benin or Botswana or Burkina Faso or Burundi  or Cameroun or Cameroon or Cape Verde or Chad or Central African Republic of Comoros or Congo or  Cote d'Ivoire or Ivory Coast or Democratic Republic of Congo or Equatorial Guinea or Eritrea or Ethiopia or  Gabon or Gambia or Ghana or Guinea or Guinea Bissau or Kenya or  Lesotho or Liberia or Madagascar or Malawi or Mali or Mozambique or Namibia or Niger or Nigeria or Rwanda or  Sao Tome or Senegal or Seychelles or Sierra Leone or Somalia or South Africa or Swaziland  or Togo or Uganda or Tanzania or Zambia or Zimbabwe or North Africa, or Algeria or Egypt or Libya or Morocco or Tunisia or western Sahara).mp. [mp=title, abstract, original title,  name of substance word, subject heading word, keyword heading word, protocol  supplementary concept, rare disease supplementary concept, unique identifier]  4. 1 and 2 and 3 |
| **EMBASE** | 1. (breast cancer* or breast neoplasm* or breast carcinoma* or breast sarcoma* or breast  tumor* or breast tumour* or breast malignanc*).mp. [mp=title, abstract, original  title, name of substance word, subject heading word, keyword heading word, protocol supplementary  concept, rare disease supplementary concept, unique identifier]  2. (survival rate or mortality rate).mp. [mp=title,  abstract, original title, name of substance word, subject heading word, keyword heading word,  protocol supplementary concept, rare disease supplementary concept, unique identifier]  3. (Africa or sub Saharan Africa or Angola or Benin or Botswana or Burkina Faso or Burundi  or Cameroun or Cameroon or Cape Verde or Chad or Central African Republic of Comoros or Congo or  Cote d'Ivoire or Ivory Coast or Democratic Republic of Congo or Equatorial Guinea or Eritrea or Ethiopia or  Gabon or Gambia or Ghana or Guinea or Guinea Bissau or Kenya or  Lesotho or Liberia or Madagascar or Malawi or Mali or Mozambique or Namibia or Niger or Nigeria or Rwanda or  Sao Tome or Senegal or Seychelles or Sierra Leone or Somalia or South Africa or Swaziland  or Togo or Uganda or Tanzania or Zambia or Zimbabwe or North Africa, or Algeria or Egypt or Libya or Morocco or Tunisia or western Sahara).mp. [mp=title, abstract, original title,  name of substance word, subject heading word, keyword heading word, protocol  supplementary concept, rare disease supplementary concept, unique identifier]  4. 1 and 2 and 3 |
| **Cochrane Library** | 1. (breast cancer* or breast neoplasm* or breast carcinoma* or breast sarcoma* or breast  tumor* or breast tumour* or breast malignanc*).mp. [mp=title, abstract, original  title, name of substance word, subject heading word, keyword heading word, protocol supplementary  concept, rare disease supplementary concept, unique identifier]  2. (survival rate or mortality rate).mp. [mp=title,  abstract, original title, name of substance word, subject heading word, keyword heading word,  protocol supplementary concept, rare disease supplementary concept, unique identifier]  3. (Africa or sub Saharan Africa or Angola or Benin or Botswana or Burkina Faso or Burundi  or Cameroun or Cameroon or Cape Verde or Chad or Central African Republic of Comoros or Congo or  Cote d'Ivoire or Ivory Coast or Democratic Republic of Congo or Equatorial Guinea or Eritrea or Ethiopia or  Gabon or Gambia or Ghana or Guinea or Guinea Bissau or Kenya or  Lesotho or Liberia or Madagascar or Malawi or Mali or Mozambique or Namibia or Niger or Nigeria or Rwanda or  Sao Tome or Senegal or Seychelles or Sierra Leone or Somalia or South Africa or Swaziland  or Togo or Uganda or Tanzania or Zambia or Zimbabwe or North Africa, or Algeria or Egypt or Libya or Morocco or Tunisia or western Sahara).mp. [mp=title, abstract, original title,  name of substance word, subject heading word, keyword heading word, protocol  supplementary concept, rare disease supplementary concept, unique identifier]  4. 1 and 2 and 3 |

**S1 Table: Literature search strategy**
