# Supplementary material for: Regional, racial, gender, and tumor biology disparities in breast cancer survival rates in Africa: A systematic review and meta-analysis
Source: PLoS One. 2019 Nov 21;14(11):e0225039. doi: 10.1371/journal.pone.0225039 (PMC6872165; doi:10.1371/journal.pone.0225039)
Supplement: S3 Table — (DOCX) [file pone.0225039.s006.docx]

| **Author** | **Country** | **Gender** | **Region** | **Race** | **Median Age** | **Sample Size** | **OS** | **n** | **Survival Early Stage%** | **Late Stage%** | **TNBC%** | **HER2+%** | **ER+%** | **PR+%** | **Quality Score** | **Surgery (%)** | **Radiotherapy (%)** | **Chemotherapy (%)** | **Endocrine therapy**  **(%)** | **Racial admixture in study (%)** |
| --- | --- | --- | --- | --- | --- | --- | --- | --- | --- | --- | --- | --- | --- | --- | --- | --- | --- | --- | --- | --- |
| Galukande (2015)^1^ | Uganda | F | East-Africa | African Black | 45 | 262 | 51.8 | 136 | 100 | 51.8 |  |  |  |  | 19 | NR | NR | NR | NR | NR |
| Gakwaya (2008)^2^ | Uganda | F | East-Africa | African Black | 45 | 297 | 56 | 166 | 74 | 39 |  |  |  |  | 13 | 75 | 76 | 29 | 60 | NR |
| Thomas (2017)^3^ | Ghana | F | West-Africa | African Black | 51 | 223 | 40 | 89 | 62 | 28 |  |  |  |  | 16 | NR | NR | NR | NR | NR |
| Anele (2014)^4^ | Nigeria | F | West Africa | African Black | 41.7 | 100 | 10 | 10 |  |  |  |  |  |  | 9 | 90 | 77 | 91 | 46 | NR |
| Cubasch (2018)^5^ | South Africa | F | South Africa | African Black | 54.4 | 602 | 60 | 361 | 78 | 39 |  |  |  |  | 22 | 74 | 52 | 67 | 46 | Black: 91, White: 4, Mixed: 3, Indian/Asia: 3 |
| Eber-Schulz (2018)^6^ | Ethiopia | F | East-Africa | African Black | 45 | 107 | 18 | 19 | 68 | 25 |  |  |  |  | 14 | 100 | 2 | NR | 27 | NR |
| Kantelhardt (2014)^7^ | Ethiopia | F | East-Africa | African Black | 43 | 1070 | 46 | 492 | 72 | 33 |  |  |  |  | 13 | 87 | NR | 83 | 81 | NR |
| Makanjuola (2014)^8^ | Nigeria | F | West-Africa | African Black | 55 | 224 | 24.1 | 54 | 46.2 | 21.7 |  |  |  |  | 14 | 100 | 45.7 | 100 | 100 | NR |
| Kene (2010)^9^ | Nigeria | F | West-Africa | African Black | 44.5 | 103 | 70.4 | 73 | 100 | 24.5 |  |  |  |  | 12 | NR | NR | NR | NR | NR |
| Ikpat (2002)^10^ | Nigeria | F | West-Africa | African Black | 42.7 | 129 | 72.1 | 93 |  |  |  |  |  |  | 11 | NR | NR | NR | NR | NR |
| Souad (2018)^11^ | Algeria | F | North-Africa | African Non-black | 52 | 472 | 45.2 | 213 |  |  |  |  |  |  | 16 | 100 | 97 | 95 | NR | NR |
| Ismail (2018)^12^ | Egypt | F | North-Africa | African Non-black | 48.8 | 50 | 57 | 29 |  |  |  |  |  |  | 14 | NR | NR | NR | NR | NR |
| Bogan (2017)^13^ | Egypt | F | North-Africa | African Non-black | 52 | 96 | 55.4 | 53 |  |  |  |  |  |  | 14 | NR | NR | NR | NR | NR |
| Aiad (2015)^14^ | Egypt | F | North-Africa | African Non-black | 53 | 84 | 54 | 45 |  |  |  |  |  |  | 13 | 100 | NR | 100 | NR | NR |
| Taha (2009)^15^ | Egypt | F | North-Africa | African Non-black | 48.8 | 21 | 46 | 10 |  |  |  |  |  |  | 10 | NR | NR | 100 | NR | NR |
| Zeeneldin (2009)^16^ | Egypt | F | North-Africa | African Non-black | 45 | 57 | 72 | 41 |  |  |  |  |  |  | 11 | 100 | 75 | 83 | 60 | NR |
| Slaoui (2016)^17^ | Morocco | F | North-Africa | African Non-black | 48.1 | 716 | 69.8 | 500 |  |  |  |  |  |  | 22 | 75 | 57 | 73 | 50 | NR |
| Ermiah (2013)^18^ | Libya | F | North-Africa | African Non-black | 45.1 | 170 | 61.3 | 104 |  |  |  |  |  |  | 16 | NR | NR | NR | NR | NR |
| Abdalla (2012)^19^ | Libya | F | North-Africa | African Non-black | 43.6 | 104 | 55.2 | 57 | 87.72 | 40.11 |  |  |  |  | 12 | 87 | 76 | 95 | 82 | NR |
| Abdalla (2012)^20^ | Libya | F | North-Africa | African Non-black | NR | 62 | 56.8 | 35 |  |  |  |  |  |  | 14 | 93 | 84 | 92 | 63 | NR |
| Ben Ahmed (2002)^21^ | Tunisia* | F | North-Africa | African Non-black | 50 | 729 | 50.5 | 368 | 71.9 | 32.5 |  |  |  |  | 12 | 86 | 56 | 73 | 47 | NR |
| Samaka (2016)^22^ | Egypt | F | North Africa | African Non-black | NR | 81 | 80 | 65 |  |  | 23.5 | 40.7 | 56.8 | 50.6 | 12 | NR | NR | NR | NR | NR |
| Derkaoui (2016)^23^ | Morocco | F | North Africa | African Non-black | 46 | 279 | 71.4 | 199 | 92.3 | 47.8 |  |  |  |  | 11 | 100 | 71 | 100 | NR | Arab: 69, Amazigh: 27, European: 2 Sub-Saharan: 2 |
| Traore (2015)^24^ | Guinea | F | West Africa | African Black | 48 | 133 | 57.9 | 77 |  |  |  |  |  |  | 8 | NR | NR | NR | NR | NR |
| Ayoade (2014)^25^ | Nigeria | F | West Africa | African Black | 48.1 | 139 | 22.3 | 31 | 60 | 17 |  | 4.6 | 36.3 | 28.9 | 14 | 41 | 41 | 89 | NR | NR |
| Zeeneldin (2013)^26^ | Egypt | F | North Africa | African Non-black | 49.5 | 5459 | 81 | 4422 |  |  |  |  |  |  | 17 | 100 | 88 | 96 | 81 | NR |
| Mohammad (2012)^27^ | Egypt | F | North Africa | African Non-black | 52.4 | 47 | 90 | 42 |  |  |  |  | 66 | 71 | 15 | NR | 79 | 87 | 75 | NR |
| Kantelhardt (2012)^28^ | Ethiopia | F | East Africa | African Black | 44.1 | 1303 | 47 | 612 | 78 | 38 |  |  |  |  | 14 | 84 | NR | 59 | 63 | NR |
| Seedhom (2011)^29^ | Egypt | F | North Africa | African Non-black | 56 | 1207 | 70 | 845 |  |  |  |  |  |  | 13 | 100 | 100 | 68 | NR | NR |
| El Mongy (2010)^30^ | Egypt | F | North Africa | African Non-black | 47.14 | 1009 | 91.4 | 922 |  |  |  |  |  |  | 13 | 100 | 92 | 80 | 65 | NR |
| Sankaranarayanan (2010)^31^ | Gambia | F | West Africa | African Black | 47 | 61 | 12 | 7 |  |  |  |  |  |  | 10 | NR | NR | NR | NR | NR |
| Sankaranarayanan (2010)^31^ | Uganda | F | East Africa | African Black | 46 | 162 | 46 | 75 |  |  |  |  |  |  | 10 | NR | NR | NR | NR | NR |
| Kaabia (2017)^32^ | Tunisia | M | North Africa | African Non-black | 54 | 44 | 26 | 11 |  |  | 36.4 |  |  |  | 9 | NR | NR | NR | NR | NR |
| Alaoui Slimani (2016)^33^ | Morocco* | M | North Africa | African Non-black | 61 | 140 | 68 | 95 |  |  |  |  |  |  | 10 | 63 | 65 | 54 | 86 | NR |
| El-Beshbeshi (2012)^34^ | Egypt | M | North Africa | African Non-black | 57.7 | 37 | 60.5 | 22 |  |  |  |  |  |  | 11 | 92 | 89 | 92 | 57 | NR |
| El-Baradie (2012)^35^ | Egypt | M | North Africa | African Non-black | 58 | 123 | 63 | 77 |  |  |  |  | 71.4 | 69.2 | 19 | 78 | 56 | 43 | 39 | NR |
| Bourhafour (2010)^36^ | Morocco | M | North Africa | African Non-black | 62 | 127 | 63 | 80 |  |  |  |  |  |  | 13 | 83 | 67 | 21 | 45 | NR |
| Ihekwaba (1993)^37^ | Nigeria | M | West Africa | African Non-black | 54 | 57 | 7 | 4 |  |  |  |  |  |  | 11 | 91 | NR | 58 | 11 | NR |
| Bouzid (2013)^38^ | Tunisia* | F | North Africa | African Non-black | 32 | 124 | 67.7 | 84 |  |  |  |  |  |  | 13 | 93 | 92 | 89 | 36 | NR |
| Khanfir (2016)^39^ | Tunisia* | F | North Africa | African Non-black | 32 | 72 | 57 | 41 |  |  |  |  |  |  | 9 | 90 | 87 | 97 | 50 | NR |
| Bakkach (2017)^40^ | Morocco | F | North Africa | African Non-black | 36 | 82 | 75.6 | 62 | 85.2 | 57.1 |  | 29.2 |  |  | 16 | 83 | 73 | 91 | 85 | Arab: 68, Amazigh: 31, European: 1 |
| Abdelkrim (2015)^41^ | Tunisia | F | North Africa | African Non-black | 24 | 25 | 85 | 21 |  |  | 24 |  |  |  | 12 | 76 | NR | NR | NR | NR |
| Kallel (2015)^42^ | Tunisia | F | North Africa | African Non-black | 31 | 83 | 66.8 | 55 | 79.2 | 42.9 |  |  |  |  | 16 | 88 | 78 | 89 | NR | NR |
| Khelifi (2012)^43^ | Tunisia | F | North Africa | African Non-black | 35 | 32 | 53.5 | 17 |  |  |  |  |  |  | 6 | 75 | 91 | 88 | 53 | NR |
| Basro (2009)^44^ | South Africa | F | South Africa | African Black | 30 | 141 | 20 | 28 |  |  |  |  |  |  | 15 | 91 | 65 | 87 | 48 | NR |
| Parag (2016^45^) | South Africa | F | South Africa | African Black | 60 | 32 | 65.6 | 21 |  |  |  |  |  |  | 10 | 66 | 41 | 59 | 72 | NR |
| Zongo (2018)^46^ | Burkina Faso | M | West Africa | African Black | 60.9 | 51 | 49 | 25 |  |  |  |  |  |  | 13 | 61 | 4 | 29 | 22 | NR |
| Ahmed (2012)^47^ | Nigeria | M | West Africa | African Black | 59 | 57 | 22.8 | 13 |  |  |  |  |  |  | 11 | 86 | 54 | 70 | 100 | NR |
| Elshafiey (2011)^48^ | Egypt | M | North Africa | African Non-black | 59 | 32 | 65.4 | 21 |  |  |  |  |  |  | 15 | 78 | 94 | 94 | 94 | NR |
| El-Habbash (2009)^49^ | Libya | M | North Africa | African Non-black | 61 | 22 | 57 | 13 |  |  |  |  |  |  | 10 | NR | NR | 71 | NR | NR |
| Ben Dhiab (2005)^50^ | Tunisia* | M | North Africa | African Non-black | 65 | 123 | 62 | 76 |  |  |  |  |  |  | 10 | 84 | NR | NR | NR | NR |
| Tabbane (1977)^51^ | Tunisia | F | North Africa | African Non-black | NR | 581 | 37.9 | 220 |  |  |  |  |  |  | 6 | NR | NR | NR | NR | NR |
| Mabula (2012)^52^ | Tanzania | F | East Africa | African Black | 45 | 348 | 21.8 | 76 |  |  |  |  |  |  | 10 | 100 | 12 | 45 | 100 | NR |
| Gobrane (2007)^53^ | Tunisia* | F | North Africa | African Non-black | NR | 470 | 61 | 287 |  |  |  |  |  |  | 11 | 63 | 65 | 50 | 10 | NR |
| Weiner (2018)^54^ | Ethiopia | F | East Africa | African Black | 42 | 573 | 15 | 86 |  |  |  |  |  |  | 15 | NR | NR | NR | NR | NR |

**S3 Table. Details of each study**

NR: not reported

OS: overall survival

n: number of patients who survived at 5 year mark

Survival Early Stage%: survival rate at early stage

Late Stage%: survival rate at late stage

TNBC%: percentage of patients with triple-negative breast cancer

HER2+%: percentage of patients with human epidermal growth factor receptor 2

(HER2)/neu positive breast cancer

ER+%: percentage of patients with estrogen receptor positive breast cancer

PR+%: percentage of patients with progesterone receptor positive breast cancer

*Non-English studies

**References**

1. Galukande M, Wabinga H, Mirembe F. Breast cancer survival experiences at a tertiary hospital in sub-Saharan Africa: a cohort study. *World journal of surgical oncology* 2015; **13**(1): 220.

2. Gakwaya A, Kigula-Mugambe JB, Kavuma A, et al. Cancer of the breast: 5-year survival in a tertiary hospital in Uganda. *British Journal Of Cancer* 2008; **99**: 63.

3. Thomas AS, Kidwell KM, Oppong JK, et al. Breast Cancer in Ghana: Demonstrating the Need for Population-Based Cancer Registries in Low- and Middle-Income Countries. *Journal of Global Oncology* 2017; **3**(6): 765-72.

4. Anele AA, Bowling M, Eckert GJ, Gonzalez E, Kipfer H, Sauder C. TREATMENT OF BREAST CANCER: IMO STATE NIGERIA VERSUS INDIANA, USA WOMEN - COMPARATIVE ANALYTIC STUDY. *Journal of the West African College of Surgeons* 2014; **4**(4): 39-69.

5. Cubasch H, Dickens C, Joffe M, et al. Breast cancer survival in Soweto, Johannesburg, South Africa: A receptor-defined cohort of women diagnosed from 2009 to 11. *Cancer Epidemiology* 2018; **52**: 120-7.

6. Eber-Schulz P, Tariku W, Reibold C, et al. Survival of breast cancer patients in rural Ethiopia. *Breast Cancer Research and Treatment* 2018; **170**(1): 111-8.

7. Kantelhardt E, Zerche P, Mathewos A, et al. Breast cancer survival in Ethiopia: a cohort study of 1,070 women. 2014; **135**(3): 702-9.

8. Makanjuola SBL, Popoola AO, Oludara MA. Radiation therapy: A major factor in the five-year survival analysis of women with breast cancer in Lagos, Nigeria. *Radiotherapy and Oncology* 2014; **111**(2): 321-6.

9. Kene TS, Odigie VI, Yusufu LM, Yusuf BO, Shehu SM, Kase JT. Pattern of presentation and survival of breast cancer in a teaching hospital in north Western Nigeria. *Oman medical journal* 2010; **25**(2): 104-7.

10. Ikpat O, Ndoma-Egba R, Collan YJEAmj. Influence of age and prognosis of breast cancer in Nigeria. 2002; **79**(12): 651-7.

11. Souad H, Zahia F, Abdelhak L, Karima S, Dalila S, Noureddine A. Descriptive study of triple negative breast cancer in Eastern Algeria. *The Pan African medical journal* 2018; **29**: 45-.

12. Ismail A, El-Awady R, Mohamed G, Hussein M, Ramadan SS. Prognostic Significance of Serum Vitamin D Levels in Egyptian Females with Breast Cancer. *Asian Pacific journal of cancer prevention : APJCP*; **19**(2): 571-6.

13. Bogan D, Meile L, El Bastawisy A, et al. The role of BRCA1-IRIS in the development and progression of triple negative breast cancers in Egypt: possible link to disease early lesion. *BMC Cancer* 2017; **17**(1): 329.

14. Aiad HA, Kandil MA, El-Tahmody MA, et al. The prognostic and predictive significance of PARP-1 in locally advanced breast cancer of Egyptian patients receiving neoadjuvant chemotherapy. 2015; **23**(8): 571-9.

15. Taha FM, Zeeneldin AA, Helal AM, et al. Prognostic value of serum vascular endothelial growth factor in Egyptian females with metastatic triple negative breast cancer. *Clinical Biochemistry* 2009; **42**(13): 1420-6.

16. Zeeneldin A, Mohamed A, Abdel H, Taha F, Goda I, AboDeef W. Survival effects of cyclooxygenase-2 and 12-lipooxygenase in Egyptian women with operable breast cancer. *Indian Journal of Cancer* 2009; **46**(1): 54-60.

17. Slaoui M, Mouh FZ, Ghanname I, Razine R, El Mzibri M, Amrani MJPo. Outcome of breast cancer in moroccan young women correlated to clinic-pathological features, risk factors and treatment: a comparative study of 716 cases in a single institution. 2016; **11**(10): e0164841.

18. Ermiah E, Buhmeida A, Khaled BR, et al. Prognostic value of bcl-2 expression among women with breast cancer in Libya. *Tumor Biology* 2013; **34**(3): 1569-78.

19. Abdalla F, Buhmeida A, Alshrad M, Salem N, Pyrhönen S, Collan YJO. Prognostic significance of DNA image cytometry in Libyan breast cancer. 2012; **83**(3): 165-76.

20. Abdalla FBE, Markus R, Buhmeida A, Boder J, SYRJÄNEN K, COLLAN YJAr. Estrogen receptor, progesterone receptor, and nuclear size features in female breast cancer in Libya: correlation with clinical features and survival. 2012; **32**(8): 3485-93.

21. Ben Ahmed S, Aloulou S, Bibi M, et al. *Santé Publique* 2002; **14**(3): 231-41.

22. Samaka RM, Younes SF. Androgen Receptor Expression in Breast Carcinoma of Egyptian Patients. *Journal of clinical and diagnostic research : JCDR* 2016; **10**(11): EC17-EC21.

23. Derkaoui T, Bakkach J, Mansouri M, et al. Triple negative breast cancer in North of Morocco: clinicopathologic and prognostic features. *BMC Women's Health* 2016; **16**(1): 68.

24. Traore B, Toure A, Sy T, et al. Five-year prognosis of breast cancer patients in Conakry Hospital University (West Africa). *Journal of Clinical Oncology* 2015; **33**(15_suppl): e12065-e.

25. Ayoade BA, Agboola AJ, Olatunji AA, Tade AO, Salami BA, Adekoya AO. Clinical characteristics and survival outcome of breast cancer in southwest Nigerian women. *Journal Africain du Cancer / African Journal of Cancer* 2014; **6**(2): 79-84.

26. Zeeneldin AA, Ramadan M, Elmashad N, Fakhr I, Diaa A, Mosaad E. Breast cancer laterality among Egyptian patients and its association with treatments and survival. *Journal of the Egyptian National Cancer Institute* 2013; **25**(4): 199-207.

27. Mohammad MA, Zeeneldin AA, Abd Elmageed ZY, et al. Clinical relevance of cyclooxygenase-2 and matrix metalloproteinases (MMP-2 and MT1-MMP) in human breast cancer tissue. *Molecular and Cellular Biochemistry* 2012; **366**(1): 269-75.

28. Kantelhardt EJ, Zerche P, Trocchi P, et al. Breast cancer in Sub-Saharan Africa: 1,000 patients with primary breast cancer in Addis Ababa followed for up to 5 years. *Journal of Clinical Oncology* 2012; **30**(15_suppl): 580-.

29. Seedhom AE, Kamal NN. Factors affecting survival of women diagnosed with breast cancer in El-Minia Governorate, Egypt. *International journal of preventive medicine* 2011; **2**(3): 131-8.

30. El Mongy M, El Hossieny H, Haggag F, Fathy R. Clinico-pathological study and treatment results of 1009 operable breast cancer cases: Experience of NCI Cairo University, Egypt. *The Chinese-German Journal of Clinical Oncology* 2010; **9**(7): 409-15.

31. Sankaranarayanan R, Swaminathan R, Brenner H, et al. Cancer survival in Africa, Asia, and Central America: a population-based study. *The Lancet Oncology* 2010; **11**(2): 165-73.

32. Kaabia O, Amine G, Nawel A, Feten H, Afraa B. MALE BREAST CANCER IN TUNISIA: EPIDEMIOLOGICAL AND CLINICAL FEATURES & PROGNOSIS FACTORS. INTERNATIONAL JOURNAL OF GYNECOLOGICAL CANCER; 2017: LIPPINCOTT WILLIAMS & WILKINS TWO COMMERCE SQ, 2001 MARKET ST, PHILADELPHIA, PA 19103 USA; 2017. p. 631-.

33. Alaoui Slimani K, Debbagh A, Sbitti Y, Errihani H, Ichou M. [Male breast cancer in Morocco: Epidemiology and prognostic factors. A report of 140 cases]. *Gynecol Obstet Fertil* 2016; **44**(11): 636-40.

34. El-Beshbeshi W, Abo-Elnaga EM. Male breast cancer: 10-year experience at mansoura university hospital in egypt. *Cancer biology & medicine* 2012; **9**(1): 23-8.

35. El-Baradie M, Salama A, Khorshid O, Ismail H, Attia G, Bahnassy AA. Egyptian male breast carcinoma: patients’ hormonal profile, management and outcome. *The Chinese-German Journal of Clinical Oncology* 2012; **11**(2): 89-98.

36. Bourhafour M, Belbaraka R, Souadka A, M'Rabti H, Tijami F, Errihani H. Male breast cancer: a report of 127 cases at a Moroccan institution. *BMC Research Notes* 2011; **4**(1): 219.

37. Ihekwaba FN. The management of male breast cancer in Nigerians. *Postgraduate Medical Journal* 1993; **69**(813): 562.

38. Bouzid N, Lahmar R, Tebra S, Bouaouina N. [Breast cancer in woman younger than 35 years in Tunisia: retrospective study about 124 cases]. *Gynecol Obstet Fertil* 2013; **41**(6): 356-60.

39. Khanfir A, Frikha M, Kallel F, et al. [Breast cancer in young women in the south of Tunisia]. *Cancer radiotherapie : journal de la Societe francaise de radiotherapie oncologique* 2006; **10**(8): 565-71.

40. Bakkach J, Mansouri M, Derkaoui T, et al. Clinicopathologic and prognostic features of breast cancer in young women: a series from North of Morocco. *BMC Women's Health* 2017; **17**(1): 106.

41. Abdelkrim SB, Fathallah K, Rouatbi R, Ayachi M, Hmissa S, Mokni M. OM.Breast Cancer in Very Young Women Aged 25 Year-Old or Below in the Center of Tunisia and Review of the Literature. *Pathology & Oncology Research* 2015; **21**(3): 553-61.

42. Kallel M, Elloumi F, Khabir A, et al. Breast cancer in young women in southern Tunisia: Anatomical study and clinical prognostic factors: About a series of 83 patients. *Reports of Practical Oncology & Radiotherapy* 2015; **20**(3): 155-60.

43. Khelifi O, Zeghal D, Jeridi S, Zouari F, Mahjoub SJIJoG, Obstetrics. M307 YOUNG WOMEN'S BREAST CANCER. 2012; **119**(S3).

44. Basro S, Apffelstaedt JP. Breast Cancer in Young Women in a Limited-Resource Environment. *World Journal of Surgery* 2010; **34**(7): 1427-33.

45. Parag Y, Buccimazza IJSSAMJ. How long are elderly patients followed up with mammography after the diagnosis of breast cancer? A single-centre experience in a developing country. 2016; **106**(7): 721-3.

46. Zongo N, Ouédraogo S, Korsaga-Somé N, et al. Male breast cancer: diagnosis stages, treatment and survival in a country with limited resources (Burkina Faso). *World Journal of Surgical Oncology* 2018; **16**(1): 4.

47. Ahmed A, Ukwenya Y, Abdullahi A, Muhammad I. Management and Outcomes of Male Breast Cancer in Zaria, Nigeria %J International Journal of Breast Cancer. 2012; **2012**: 6.

48. Elshafiey MM, Zeeneldin AA, Elsebai HI, et al. Epidemiology and management of breast carcinoma in Egyptian males: Experience of a single Cancer Institute. *Journal of the Egyptian National Cancer Institute* 2011; **23**(3): 115-22.

49. El-Habbash MM, Alwindi AA. Male breast cancer in Tripoli, Libya. *Saudi medical journal* 2009; **30**(8): 1060-2.

50. Ben Dhiab T, Bouzid T, Gamoudi A, et al. [Male breast cancer: about 123 cases collected at the Institute Salah-Azaiz of Tunis from 1979 to 1999]. *Bulletin du cancer* 2005; **92**(3): 281-5.

51. Tabbane F, Muenz L, Jaziri M, Cammoun M, Belhassen S, Mourali NJC. Clinical and prognostic features of a rapidly progressing breast cancer in Tunisia. 1977; **40**(1): 376-82.

52. Mabula JB, Mchembe MD, Chalya PL, et al. Stage at diagnosis, clinicopathological and treatment patterns of breast cancer at Bugando Medical Centre in north-western Tanzania. 2012; **14**(4).

53. Abdallah MB, Achour N, Hsairi MJEMHJ. Pronostic du cancer du sein à l'Institut de Carcinologie Salah Azaiez de Tunis. 2007; **13**(2): 309.

54. Weiner CM, Mathewos A, Addissie A, et al. Characteristics and follow-up of metastatic breast cancer in Ethiopia: A cohort study of 573 women. 2018; **42**: 23-30.
